# Epidemiology of non-trauma orthopedic conditions among inpatients admitted at a tertiary teaching and referral hospital in Kenya: A chart review

**DOI:** 10.1371/journal.pone.0303898

**Published:** 2024-06-17

**Authors:** Maxwell Philip Omondi

**Affiliations:** Orthopedic Unit, Department of Surgery, University of Nairobi, Nairobi, Kenya; National Trauma Research Institute, AUSTRALIA

## Abstract

Non-traumatic orthopedic conditions are pathological conditions involving musculoskeletal system that includes muscles, tendons, bone and joints and associated with frequent medical and surgical care and high treatment costs. There is paucity of information on the pattern of non-traumatic orthopedic conditions in low and middle income countries. The purpose of this study was to determine the epidemiology of non-traumatic orthopedic conditions among inpatients at the Kenyatta National Hospital in Kenya. This was a cross-sectional study with a sample of 175 charts reviewed. Approximately, 70.3% of the inpatients were aged between 25 to 64 years of age with the mean age of 39.97 years (STD 18.78). Ever married tended to be older 53.5 (95% CI: 46.8–60.2) years than other marital statuses. Approximately, 60.6% were males, 38.9% had comorbidities and 49.1% were casuals or unemployed. All inpatients were Kenyans with Nairobi County comprising 52.6% of all inpatients. Approximately, 77.7% were self-referrals. The commonest non-trauma orthopaedic conditions were infection and non-union (35.4%) and spinal degenerative diseases (20.60%) and the least was limb deformities (1.70%). Compared to females, males were 3.703 (p<0.001) times more likely to have infection and non-union. Patients with primary, secondary and tertiary education were 88.2% (p<0.001), 75.6% (p<0.001) and 68.1% (p = 0.016) less likely to have infection and non-union compared to those with no or preschool education. Widows were 8.500 (p = 0.028) times more likely to have spinal degenerative disease than married. Males were 70.8% (p = 0.031) less likely to have osteoarthritis than females. Inpatients with secondary education were 5.250 (p = 0.040) times more likely to have osteoarthritis than those with no or preschool education. In conclusion, majority of inpatients were young and middle aged adults. Infection and non-union and spinal degenerative diseases were the most common non-trauma orthopedic conditions. While males and those with low education were more likely to have infection and non-union, married were more likely to have spinal degenerative disease. Osteoarthritis was more likely among female admissions.

## Background

Non-traumatic orthopedic conditions are pathological conditions involving muscles, tendons, bone and joints in both appendicular and axial skeleton that could be congenital, developmental or acquired in nature. They are associated with significant morbidity that results in reduced productivity, chronic absenteeism at work, disability and generally high health care costs due to frequent medical and surgical care needed [1].

Non-traumatic orthopedic conditions such as low back pain, compressive neuropathies, and degenerative joint disorders affect mostly adults while orthopedic infections and musculoskeletal tumours affect all populations–young and the old. Non-traumatic orthopedic conditions are generally common morbidities at health facilities [2].

The most frequently occurring non-traumatic orthopedic conditions are osteoarthritis, back and neck pain, fragility fractures, and systemic inflammatory conditions such as rheumatoid arthritis. These are usually chronic disabling conditions with significant morbidity and compromised quality of life. They are more common in older populations, especially between 65 and 74 years of age [3].

A study on epidemiological patterns of traumatic musculoskeletal injuries and non-traumatic disorders in Japan Self-Defense Forces revealed that the most common were lumbar, tendon and joint disorders and the incidence increases with advancing age [4]. This study was comprised both outpatients and inpatients with musculoskeletal injuries in Japan Self-Defense Forces.

Studies on orthopedic admissions at a tertiary health facilities in Northern Tanzania and Nepal showed infection was the commonest non-trauma orthopedic condition comprising about 7% - 12% of all admissions [5, 6]. These studies looked at both trauma and non-trauma–related injuries admissions and did not look at the distribution and possible risk factors for non-trauma orthopedic admissions at the tertiary heath facilities.

Therefore there is a lack of literature on the occurrence of non-traumatic orthopedic conditions in inpatient settings in low and middle income countries, Kenya inclusive. The purpose of this study was to determine the epidemiology of non-traumatic orthopedic conditions among inpatient admissions in a tertiary health facility in Kenya. This will be important in hospital resource allocation and planning in the management of non-trauma orthopedic conditions in KNH and similar tertiary health facilities in Kenya and in the region.

## Methods and materials

### Study design

This was a retrospective cross-sectional study design.

### Study area

The KNH is the largest teaching and referral hospital in East and Central Africa. KNH Orthopedic Wards were used as the study area. The KNH is based in Upperhill, Nairobi, the capital city of Kenya. It is located approximately 5 km from the city center. KNH has a bed capacity of 1,800, over 6,000 staff members, 50 wards, 22 outpatient clinics, 24 theaters (16 specialized) and an Accident & Emergency Department [7]. Of the 1800 bed capacity, 96 beds are allocated to orthopedic wards. KNH is a 10-floor storied building complex, and the orthopedic wards are located on the 6^th^ floor; however, orthopedic admissions to private wings occurred on the 9^th^ and 10^th^ floors. Orthopedic patients with other comorbidities were also admitted to other wards in the KNH.

### Study duration

The study duration was from 1^st^ February 2021 to 31st December 2021. Data abstraction was done from 1^st^ January 2022 to 31^st^ March 2022.

### Study population

Orthopedic inpatient admissions at the KNH in 2021.

### Inclusion criteria

All non-trauma orthopedic conditions admitted at KNH from 1^st^ February 2021 to 31^st^ December, 2021.

### Sample size

Fisher’s formula was used to calculate the sample size [8, 9]

$${\text{n}=\text{Z}}^{\text{2}}\times \text{p}\left(\text{1-p}\right){)/\text{d}}^{2}$$

The sample size was 175 charts after adjusting for study population fewer than 10,000.

### Recruitment and sampling procedures

Three (3) research assistants (RAs) were recruited to collect and abstract patient data from patient files. The RAs were health care workers with a diploma in Orthopedic Trauma and with some experience in research data collection. The Principal Investigator (PI) was the research coordinator for the data collection. Orthopedic and trauma admissions were identified from the a) admission desk of the Health Information System at the KNH Accident and Emergency Unit (A&E), b) KNH Orthopedic Outpatient Clinic Records (OC), and c) KNH Corporate Outpatient Care (COC). The data were subsequently recorded in a logbook. This logbook served as a master register for all inpatients admitted and therefore the sampling frame for the study. All admissions were logged into the logbook from the admission books stationed at these three (3) services points. The proportional population-to-size (PPS) was subsequently used to determine the number of patients sampled per month from each of these three service points so that the sample size would be representative of the admissions by month from each of these three orthopedic admission entry points (Table 1).

**Table 1:** Non-trauma orthopedic patients admitted to KNH stratified by point of admission, 2021.

Month of the year, 2021`	Point of admission			
	A&E	Clinic	COC	Total
**February**	6	6	4	16
**March**	3	2	7	12
**April**	3	2	7	12
**May**	7	2	7	16
**June**	4	2	6	12
**August**	7	6	13	26
**September**	4	4	7	15
**October**	6	5	5	16
**November**	7	6	17	30
**December**	5	4	11	20
**Total**	52	39	84	175

The three (3) RAs were reporting to and working under the direction of the PI. The RAs were trained for two (2) days by the PI on the research protocol, data collection tools, and data collection procedures; pilot testing of the data collection tools was also performed before the actual data abstraction.

### Variables

The variables extracted were admission date (dd/mm/yyyyy), age, sex, and marital status, religion, occupation, education level, referring health facility tier, nature of admission, type of admission, non-trauma orthopedic condition, Comorbidities.

### Data collection procedures

The data were collected through a data abstraction form from the patient files. Missing data were collected by calling patients or their guardians using the mobile phones provided in the patients charts after obtaining their verbal consent.

#### Data abstraction form

The three (3) RAs were trained on the data abstraction using a data abstraction form as per the research protocol (S1 Text). The PI reviewed all the completed abstraction forms for completeness and accuracy daily during the entire data collection period and provided regular feedback to the RAs in a timely manner to ensure data quality and compliance with the study protocol. All the completed and verified data abstraction forms were then collected and filed by the PI at the end of every week in a locked cabinet.

### Data management and analysis

The data abstraction tool was designed to collect both quantitative and qualitative data. While the author had access to information that could identify individual participants during or after data collection, for anonymity and confidentiality purposes, the data abstraction tools were marked only with the participant study numbers, and no names were used. The data were entered into a password-protected Redcap database (version 7.2.2; Vanderbilt, Nashville, TN, USA) maintained by the KNH Medical Research Department. The data were exported and then analyzed using SPSS version 27.0 (version 25.0; IBM, Ltd., North Carolina, USA) (S1 File). Descriptive statistics such as frequencies were calculated, while inferential statistics namely Pearson’s chi-square test and logistic regression were used. The calculations were performed at the 95% confidence level.

### Ethics approval and consent to participate

The study protocol was presented to the UoN/KNH Ethics and Research Committee and was granted ethical approval (ERC No: P852/10/2021). Administrative approval was also granted by the KNH Medical Research Department and KNH Orthopedics Department. Written informed consent was obtained from the KNH Medical Research Department to have access to the patient’s health records in the Health Information Office (Room 19). Informed verbal consent was obtained from the discharged patients who had missing information in their charts as per the UoN/KNH ethics regulations. For minors, verbal consent was obtained from parents or guardians for charts with missing values. The verbal consent was obtained over the phone. The PI and RAs took into account the visual and auditory privacy during the telephone conversation with the discharged patients, parents or guardians.

## Results

### The socio-demographic profile of the sample population

A total of 175 charts were extracted and analyzed.

Approximately, 70.3% of the inpatient admissions were aged between 25 to 64 years of age, with children representing 11.4% and elderly being 8.6%. The mean age was 39.97 years (STD 18.78). Approximately, 60.6% of inpatients admissions were males. About 55.5% had secondary or tertiary education. About 56% were married and 12.6% were minors. Approximately, 49.1% were casuals or unemployed. About 54.3% of admissions are self-referrals. All admissions were Kenyans with Nairobi County comprising 52.6% of all inpatient admissions and this increased to 75.5% if Nairobi County and its environs are included. About 70.3% of the admissions were elective and admitted through the KNH clinic and COC. An emergency admission through accident and emergency department was 29.7%. Approximately, 77.7% of the inpatient admissions were self-referrals with the remaining 22.3% were health facility referrals. Approximately, 13.1% and 24.6% were smokers and took alcohol, respectively, at the time of admission to KNH. About 38.9% had a comorbidity of which the most prevalent were hypertension (41.2%), Diabetes Mellitus (23.5%), HIV (13.2%), Allergy/Asthma (7.4%) and Gastritis (5.9%) (Table 2).

**Table 2:** Socio-demographic profile of the sample population at KNH, 2021.

Variable	Categories	Frequency, n (%)
**Age**	0–14 years	20 (11.4%)
	15–24 years	17 (9.7%)
	25–64 years	123 (70.3%)
	Above 65 years	15 (8.6%)
**Sex**	Female	69 (39.4%)
	Male	106 (60.6%)
**Education**	None & Preschool	14 (8.0%)
	Primary	58 (33.1%)
	Secondary	40 (22.9%)
	Tertiary	57 (32.6%)
	aMissing	6
**Marital status**	Married	98 (56.0%)
	Minor	22 (12.6%)
	Ever married	18 (10.3%)
	Single	37 (21.1%)
**Occupation**	Business	27 (15.4%)
	Casual	45 (25.7%)
	Employed	41 (23.4%)
	Other	16 (9.1%)
	Unemployed	41 (23.4%)
	aMissing	5
**Country**	Kenya	175 (100%)
**County**	Others	42 (24.0%)
	Kajiado	11 (6.3%)
	Kiambu	19 (10.9%)
	Nairobi	92 (52.6%)
	Others ‐ Eastern	10 (5.7%)
	aMissing	1
**Type of admission**	Elective	123 (70.3%)
	Emergency	52 (29.7%)
**Point of admission**	A&E	52 (29.7%)
	Clinic	39 (22.3%)
	COC	84 (48.0%)
**Type of referring health facility**	Self-referrals	95 (54.3%)
	Government health facilities	19 (10.9%)
	Private health facilities	20 (11.4%)
**Nature of admission**	Self-referrals	136 (77.7%)
	Facility referral	39 (22.3%)
**Smoking**	Yes	23 (13.1%)
	No	138 (78.9%)
	aMissing	14
**Alcohol**	Yes	43 (24.6%)
	No	116 (66.3%)
	aMissing	16
**Comorbidities**	No	107 (61.1%)
	Yes	68 (38.9%)

^a^Missing data were excluded from the computation of frequency distribution. Ever married includes widow, separated and divorced.

### Patterns of non-trauma orthopedic conditions

Ever married tended to be older 53.5 (95% CI: 46.8–60.2) years than other marital statuses followed by married and single patients. Single are mostly young adults (Table 3).

**Table 3:** Marital status and mean age for non-trauma orthopaedic inpatient admissions at KNH, 2021.

Marital categories	Mean age
**Married**	48.5 (95% CI: 45.8–51.1) years
**Minor**	8.1 (95% CI: 5.9–10.3) years
**Ever married**	53.5 (95% CI: 46.8–60.2) years
**Single**	29.9 (95% CI: 26.5–33.3) years

95% confidence intervals were calculated from the mean age. Ever married includes widowed, widower, separated and divorced.

**Fig 1:**
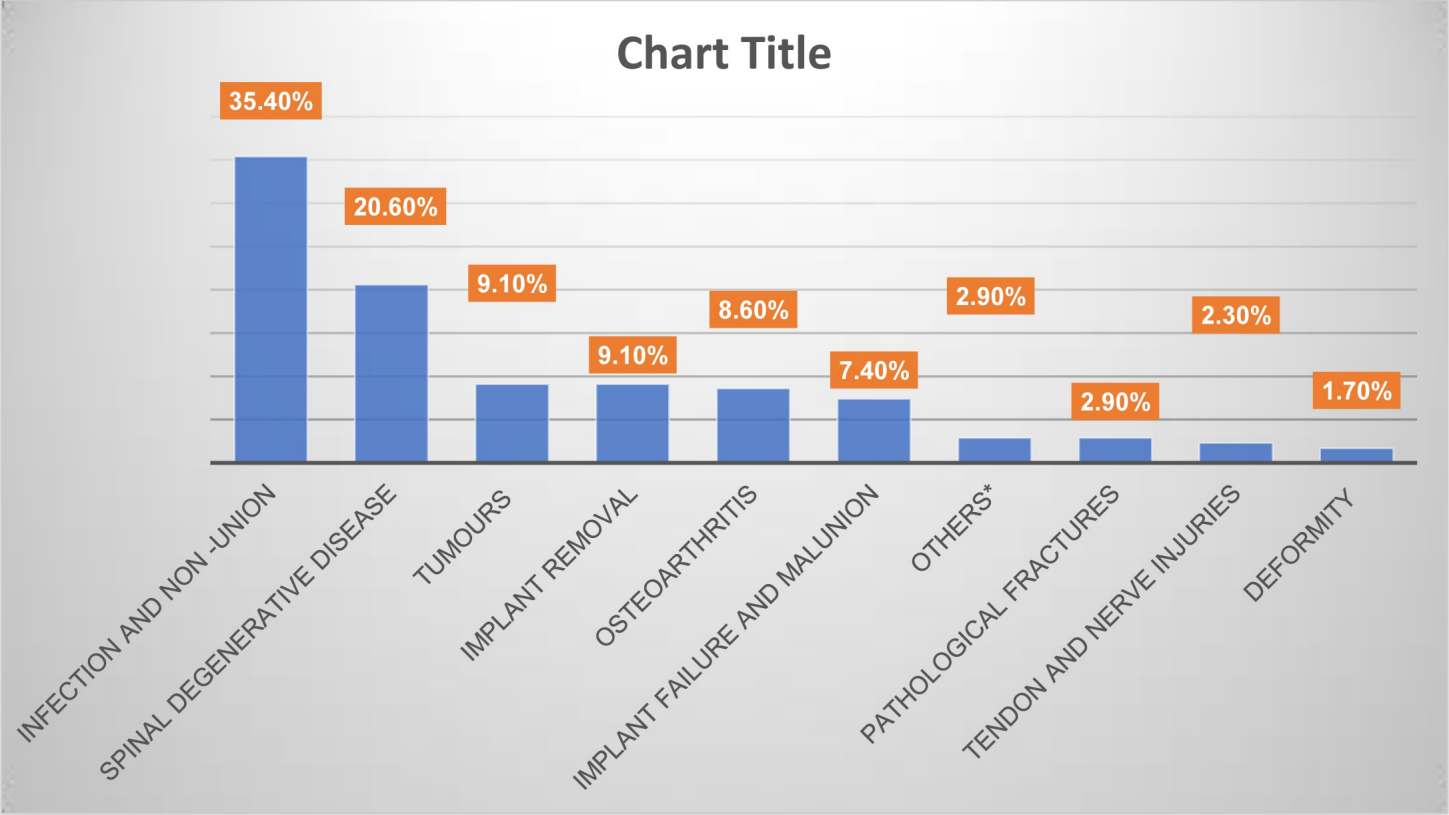
Graphical representation of common non-traumatic orthopaedic conditions admitted at KNH, 2021. Frequency distribution tally for common non-traumatic orthopaedic conditions (n = 175).

The commonest non-trauma orthopaedic admissions were infection and non-union, spinal degenerative disease comprising 56% of admissions. These were followed by tumors, implant removal, osteoarthritis and implant removal and mal-union. Pathological fractures, tendon and nerve injuries and deformity conditions were the least common admissions (Fig 1).

#### Infection and non-unions

Compared to females, males were 3.703 (p<0.001) times more likely to have infection and non-union. Patients with primary, secondary and tertiary education were 88.2% (p<0.001), 75.6% (p<0.001) and 68.1% (p = 0.016) less likely to have infection and non-compared compared to those with no or preschool education. Minors were 2.776 times more likely to have infection and non as compared to married patients (p = 0.011). Patients with “Other” occupation were 2.922 (p = 0.034) times more likely to have infection and non-union than businessmen or women. Emergency admissions were 9.669 (p<0.001) times more likely to have infection than elective admissions. Admissions through clinic and COC were 92.6% (p<0.001) and 70.2% (p = 0.008) less likely to have infection and non-union, respectively, than Accident and Emergency admissions. Admissions through the government and private health facilities referrals were 77.7% (p = 0.023) and 87.7% (p = 0.008) less likely to have infection and non-union, respectively, than self-referrals (Table 4).

**Table 4:** Cluster differences in non-trauma orthopedic conditions according to logistic regression analysis of inpatients in KNH 2021.

		Infection and non-union OR (p-value)	Spinal degenerative Disease OR (p-value)	Tumors OR (p-value)	Osteoarthritis OR (p-value)
Sex	Female	1.0	1.0	1.0	1.0
	Male	3.703 ***(p<0*.*001)***	0.538 (p = 0.097)	0.622 (p = 0.367)	0.292 ***(p = 0*.*031)***
Education	None and preschool	1.0	1.0	1.0	1.0
	Primary	0.118 ***(p = 0*.*001)***	2.143 (p = 0.353)	-	2.437 (p = 0.418)
	Secondary	0.244 ***(p = 0*.*001)***	1.714 (p = 0.241)	0.641 (p = 0.429)	5.250 ***(p = 0*.*040)***
	Tertiary	0.319 ***(p = 0*.*016)***	1.071 (p = 0.884)	4.588 (p = 0.166)	3.562 (p = 0.117)
Marital status	Married	1.0	1.0	1.0	1.0
	Minor	***2*.*776 (p = 0*.*011)***	***0*.*221 (p = 0*.*019)***	2.255 (p = 0.246)	0.182 (p = 0.106)
	Ever married	1.847 (p = 0.266)	1.211 (p = 0.998)	0.412 (p = 0.227)	0.583 (p = 0.708)
	Single	1.319 (p = 0.631)	***0*.*176 (p = 0*.*027)***	0.970 (p = 0.973)	0.249 (p = 0.999)
Occupation	Business	1.0	1.0	1.0	1.0
	Casual	1.030 (p = 0.953)	0.309 (p = 0.087)	6.303 (p = 0.092)	1.333 (p = 0.818)
	Employed	0.885 (p = 0.780)	0.378 (p = 0.127)	5.212 ***(p = 0*.*045)***	0.718 (p = 0.724)
	Other	2.922 ***(p = 0*.*034)***	0.295 (p = 0.054)	2.242 (p = 0.219)	0.249 (p = 0.096)
	Unemployed	2.125 (p = 0.252)	0.324 (p = 0.150)	3.636 (p = 0.243)	0.359 (p = 0.328)
Type of admission	Elective	1.0	1.0	1.0	1.0
	Emergency	9.669 ***(p<0*.*001)***	0.227 ***(p = 0*.*008)***	0.518 (p = 0.321)	0.338 (p = 0.164)
Point of admission	A&E	1.0	1.0	1.0	1.0
	Clinic	0.074 ***(p<0*.*001)***	6.667 ***(p<0*.*001)***	1.719 (p = 0.440)	3.767 (p = 0.093)
	COC	0.298 ***(p = 0*.*008)***	6.667 ***(p = 0*.*003)***	0.716 (p = 0.581)	2.788 (p = 0.197)
Type of referring health facility	Self-referrals	1.0	1.0	1.0	1.0
	Government health facilities	0.223 ***(p = 0*.*023)***	2.303 (p = 0.120)	2.218 (p = 0.281)	2.528 (p = 0.305)
	Private health facilities	0.123 ***(p = 0*.*008)***	2.872 (p = 0.179)	1.500 (p = 0.678)	0.944 (p = 0.957)
Comorbidities	No	1.0	1.0	1.0	1.0
	Yes	0.891 (p = 0.723)	1.450 (p = 0.321)	0.939 (p = 0.907)	2.568 (p = 0.088)

OR refers to odds ratio. P-value calculated at 5% level of significance. 1.0 refers to reference group. A&E refers to the Accident and Emergency. COC refers to the Corporate Outpatient Clinic.

#### Spinal degenerative disease

Minors and singles were 77.9% (p = 0.019) and 0.176 (p = 0.027) less likely to have spinal degenerative disease compared to married patients, respectively. Emergency admissions were 77.3% (p = 0.008) less likely to have spinal degenerative disease than elective admissions. KNH clinic and COC admissions were 6.667 (p<0.001) and 6.667 (p = 0.003) times more likely to have spinal degenerative disease, respectively, than admissions through Accident and Emergency department (Table 4).

#### Tumours

Employed inpatient admissions were 5.212 (p = 0.045) times more likely to have tumors compared with businessmen and women. Emergency admissions were 48.2% less likely to have tumors than elective admissions. However, this was not statistically significant (Table 4).

#### Osteoarthritis

Males were 70.8% (p = 0.031) less likely to have osteoarthritis than females. Inpatients with secondary education were 5.250 (p = 0.040) times more likely to have osteoarthritis than those with no or preschool education (Table 4).

## Discussion

### Socio-demographic profile of the sample population

The study revealed that majority of admissions was aged between 25 to 64 years, with mean age of about 40 years and married or single. This compares with a study done in Malawi that showed musculoskeletal impairment was most common among 31–60 years of age with prevalence increasing with increasing age and majority are married or single [10, 11]. The non-traumatic musculoskeletal disorders are common among middle aged and becomes common with advancing age.

Overall, all admissions were Kenyans with Nairobi County comprising about half of the admissions. Nairobi County and its environs comprise about three-fourths of inpatients admissions at KNH. This finding is similar to studies performed in South Africa, Tanzania, Malawi and Iran, which revealed that the majority of patients admitted to tertiary hospitals were from regions co-located with the hospital [12–16]. This may be because of the proximity of the hospitals to patients’ area of residence and because patients, relatives and friends prefer their loved ones to be admitted closer home where they can easily visit their sick. About 4 out of every 10 admissions had comorbidity with the most prevalent ones being hypertension, diabetes mellitus, HIV and allergy/asthma. This compares with a study on prevalence and patterns of comorbidity among musculoskeletal disorders that revealed the prevalent comorbidities were hypertension and diabetes [17]. These are lifestyle diseases that are common with advancing age. However, these comorbidities had no influence on the occurrence of non-trauma orthopedic conditions in the current study.

### Patterns of non-trauma orthopedic conditions

Over two-thirds of the admissions were electives, admitted mostly through the clinics. This parallels a study done in Brazil and UK that demonstrated elective admissions dominated non-traumatic orthopedic conditions [18–20]. A big proportion of non-traumatic orthopedic conditions are considered non-urgent medical conditions that are mostly managed electively and are usually seen and admitted from the outpatient clinics rather than accident and emergency department.

Over three-fourths of the admissions were self-referrals. This is in tandem with a longitudinal study done in Netherlands that revealed self-referral of patients with non-traumatic musculoskeletal conditions is common [21].

About one-tenth and a quarter of the inpatients admissions were smokers and took alcohol, respectively. This compares with a study done in Netherlands that showed musculoskeletal impairment is common among smokers [22]. Smoking and alcohol depresses body immunity and makes them prone to orthopedic infections, non-union, orthopedic tumours and pathological fractures.

This study showed that infection and non-union together with spinal degenerative disease were the most common non-trauma orthopaedic conditions admitted at KNH. This agrees with a study done in Nepal and Tanzania that showed infection is the most common form of non-trauma orthopaedic admissions [5, 6, 14]. However, it contrasts with a study done in India that showed spine conditions and implant removal were the most prevalent non-trauma orthopaedic conditions and infection was among the least common conditions [23]. This could be because Warangal Hospital in India is a multi-speciality teaching hospital that could be admitting patients of higher socio-economic status than is the case with the KNH. The study also contrasts with one done in PCEA Kikuyu Mission Hospital in Kenya that showed Osteoarthritis was the most common reason for non-trauma orthopaedic admission followed by limb deformities, infection and removal of implants [24]. This could be because the PCEA Kikuyu Mission Hospital had specialists’ orthopaedic surgeons for arthroplasty and tended to get referrals from other facilities for patients with hip and knee osteoarthritis.

#### Infection and non-union

The study showed that infection and non-union were more common among males inpatients. This is in tandem with studies in done USA, Pakistan, Iran and Germany that revealed men are more prone to bloodstream and surgical site infections including orthopaedic infections [25–28]. However, it differs with another study done in Germany that revealed no gender specific differences in surgical site infections were found in orthopaedic surgery. But it showed that if all specialities procedures were considered women had a lower rate of surgical site infections [29]. The differences in sex preposition to surgical site infections could be due to hormonal and immune response differences between male and female that accounts for lower occurrence of surgical site infections among females as compared to males. It is an established knowledge from numerous studies that infections are a major cause of fracture non-union and hence a lower occurrence of fracture non-union among female orthopaedic admissions, given the fact that they have low risk of surgical site infections.

Patients with higher education tended to have less infection and non-union than were those with no or preschool education. This parallels study done in Pakistan that revealed lower education and lower socio-economic status was strongly associated with risk of surgical site infections [28].

Minors were about three (3) times more likely to have infection and non-fracture union as compared to married patients. The mean age of minors was 8.1 years while married patients were 48.5 years. This means that younger age was associated with the risk of infection and hence fractures non-union. This is in tandem with a number of studies that have shown extremes of age–the very young and the elderly above 65 years ‐ are significantly associated with infections including surgical site infections [30–39]. This could be due to increased vulnerability resulting from immunosenescence, malnutrition as well as a variety of physiological and anatomical age-associated factors [31, 40, 41]. Married patients in this study were not elderly and hence low risk of infections and hence fracture non-union. The study also revealed that orthopaedic infections were associated with emergency admissions, admissions through accident and emergency department and self-referred patients. This is in tandem with studies done in UK that showed orthopaedic infections are associated with emergency admissions [42, 43]. Musculoskeletal infections have always been treated as one of the orthopaedic emergencies [44].

#### Spinal degenerative disease

Minors and singles were 77.9% (p = 0.019) and 0.176 (p = 0.027), respectively, less likely to have spinal degenerative disease compared to married patients. This study showed that spinal degenerative diseases were less likely among the young (minors and singles) as compared to the older age group (married patients). Minors had a mean age of 8.1 years, singles had a mean age of 29.9 years while married patients had a mean age of 48.5 years and the study therefore showed older patients were more prone to spinal degenerative disease as compared to younger patients‥ This agrees with a study done in China and Turkey that showed advancing age is associated with lumbar disc degeneration [10, 22, 45–50]. Also study in Italy revealed that chronic musculoskeletal degenerative conditions are associated with older age groups [10, 22].

The study also demonstrated that spinal degenerative disease were mostly elective admissions through the clinics. This is in tandem with a study done in Canada, USA that showed majority of degenerative spinal conditions were elective cases [51–53]. This is because they are degenerative conditions are common with advancing age and are usually chronic and non-urgent in nature.

#### Tumours

The study showed that inpatients admissions that were employed were more likely to have tumors. However, this study did not elicit the nature of employment of the inpatient admissions. Nevertheless, association between occupation and development of cancers has been documented in many studies over the century and has sometimes led to the identification of concerned carcinogens. This study agrees with a study done in USA that showed bone tumors are associated with those employed as blacksmiths, machine operators [54, 55].

#### Osteoarthritis

The study showed that females were more likely to have osteoarthritis. Sex bias in the prevalence and severity of osteoarthritis have been known for many years with females affected more than males with incidence increasing significantly after menopause [56–59]. Studies have been done that shows women have thinner articular cartilage than men and more predisposed not only to arthritis but also to severe forms of osteoarthritis.

The study also revealed that osteoarthritis was associated with secondary education. Several studies have shown low education, low socio-economic status is associated with increased prevalence of osteoarthritis [22, 60, 61]. This may be because low socio-economic status and low education levels are associated with occupation with strenuous activity level.

This study had several limitations. First, there is a possible effect of the COVID-19 pandemic on patients seeking health services at the KNH. The movement restrictions imposed during the COVID-19 pandemic could skew access to services at the KNH. This was mitigated by ensuring that the data collection period covered the COVID-19 period, during which inter-county movement restrictions were lifted by the Kenyan government. Second, this was a retrospective cross-sectional study design and hence was weak in determining causality. Third, there were missing data from the patient charts. This was mitigated by increasing the sample size by 10%. Fourth, this was a single health facility study and therefore may lack external validity. However, the tertiary health facility is the largest referral and teaching health facility in Kenya with a large catchment population and likely generalized to the Kenyan health facilities. Despite these limitations, given the dearth of information on non-trauma orthopedic conditions in Kenya, these findings could be used not only to help policy makers formulate public health preventive measures but also to help hospital management teams apportion resources appropriately for the care of these non-traumatic orthopedic conditions.

## Conclusion

Majority of non-trauma orthopedic conditions inpatient admissions were young and middle aged adults who were either casuals or unemployed. Most of them were from within Nairobi County and its environs. Infection and non-union and spinal degenerative diseases were the most common non-trauma orthopedic conditions. Males and low education were more likely to have infection and non-union. Minors and singles were younger than other marital statuses and were less likely to have spinal degenerative diseases. Osteoarthritis was more likely among females than men inpatient admissions. Cardiovascular risk factors were the most prevalent comorbidities but they had no influence on occurrence of non-traumatic orthopedic conditions.

### Recommendations

1. KNH and other similar tertiary health facilities should apportion more resources and supplies towards infection prevention and treatment in orthopedic wards;
2. KNH and the county governments should allocate more resources towards training of health care workers, patients and caregivers on infection prevention measures, early detection and treatment of orthopedic infections;
3. Aggressive infection prevention measures be instituted for emergency admissions and self-referred patients;
4. Further studies need to be done to determine the nature of employments and their predisposition for tumours to help in developing orthopedic tumours prevention guidelines;

## Supporting information

S1 File SPSS dataset Feb 2024. (SAV)

S1 Text Data abstraction tool. (DOCX)

S2 Text STROBE statement—Checklist of items that should be included in reports of *cross-sectional studies*. (DOC)

S3 Text Human participants research checklist. (DOCX)

## List of abbreviations

COVID-19: Coronavirus Disease
ERC: Ethics Review Committee
KNH: Kenyatta National Hospital
UoN/KNH: University of Nairobi/Kenyatta National Hospital
RA: Research Assistant
SPSS: Statistical Package for the Social Sciences
SOPs: Standard operating procedures
USA: United States of America
PCEA: Presbyterian Church of East Africa
PI: Principal Investigator
