# Supplementary material for: Epidemiology of non-trauma orthopedic conditions among inpatients admitted at a tertiary teaching and referral hospital in Kenya: A chart review
Source: PLoS One. 2024 Jun 17;19(6):e0303898. doi: 10.1371/journal.pone.0303898 (PMC11182543; doi:10.1371/journal.pone.0303898)
Supplement: S1 Text — (DOCX) [file pone.0303898.s002.docx]

**Data Abstraction Tool**

Serial No………………..……………

Admission ward………………………

Date of admission ………………….…

Day of the week………..…..…………

Month……..………………………..

Year………………………………..

Area of accident……………………N/A…..

Time of accident……………………N/A….

Time of accident…………..…………N/A.

Date of accident/injury…………………N/A

The month of accident ……………….N/A

Year of accident……………………..N/A

**EVENTS SURROUNDING ADMISSION (Applicable for accident/injury victims)**

1. Nature of admission a) Walk-ins b) facility referral
2. Previous admission for current orthopedic condition Yes [ ] No [ ]
3. If yes, which facility? Private [ ] Public [ ]
4. You have a referral letter …………………………… Yes [ ] No [ ]
5. If yes, from which health facility……………………………………..
6. Point of admission …………….. A&E [ ] Clinic [ ]
7. Mechanism of injury
   1. RTA b. Assault c. Fall d. Gunshot e. Stab wounds f. Others

Explain……………………………………………………………………………………………………………………………………………………………………………………………………………………………………………………………………………………

**REASONS FOR SEEKING SERVICES TO KNH**

1. A) Which facility is a major health facility closest to you (closest to the area of accident)?..............................................................................................

B) Why did you bypass the facility? a) Walk-in b) self-referral c) facility referral

- Human resource ………………………………………………………………………………………………………………………………………………………………………………………………………………………………………………………………………………………………
- Infrastructure……………………………………………………………………………………………………………………………………………………………………………………………………………………………………………………………………………
- Equipment and implants availability………………………………………………………

……………………………………………………………………………………………………………………………………………………………………………………………………………………………………………………………………………………………

- Patients factors & preference………………………………………………………………

……………………………………………………………………………………………………………………………………………………………………………………………………………………………………………………………………………………………………………………………………………………………………………………………..

- Financial……………………………………………………………………………………………………………………………………………………………………………………………………………………………………………………………………………………………………………………………………………………………………………………
- Not well defined……………………………………………………….
- Others………………………………………………………………………………………………………………………………………………………………………………………………………………………………………………………………………………………

**SOCIODEMOGRAPHIC**

1. a) Country of Residence ……………………...b) County of Residence…………..………….
2. a) Sub-county of Residence……………………b) Area/Estate of residence…………………
3. Type of admission a) emergency b) elective
4. Mode of payment
   1. Insurance (specify - NHIF, CFC, Britam, NEMIS, CIC, etc)……………………….
   2. Cash payment
5. Age (in years)………..………
6. Sex………….. Male [ ] Female [ ]
7. Marital status…

Married [ ] Divorced [ ] Separated [ ] Widowed [ ] Widower [ ] Single [ ]

1. Religion……Protestant [ ] Pentecostal [ ] Catholic [ ] Muslim [ ] Hindu [ ] Atheist [ ]
2. Occupation……

Farmer [ ] Businessman/woman [ ] Casual [ ] Unemployed [ ] Employed [ ] Student [ ] Motorbike rider [ ] Driver [ ] N/A [ ]

1. Education level… Tertiary [ ] Secondary [ ] Primary [ ] Nursery [ ] Kindergarten [ ] pre-school [ ] None [ ]

**TYPES**

1. Type of Orthopaedic injury
   1. Open [ ] closed [ ]
   2. If Open – Gustillo-Anderson classification Gustilo I [ ] Gustilo II [ ]

Gustilo IIIa [ ] Gustillo IIIb [ ] Gustllo IIIc [ ]

- 1. Trauma Orthopaedic Injuries – AO/OTA classification & ICD version 10
     1. Humerus [ ]
     2. Radius/Ulnar [ ]
     3. Hand [ ]
     4. Femur fractures [ ]
     5. Tibia/Fibula [ ]
     6. Foot [ ]
     7. Pelvic Fractures [ ]
     8. Acetabular [ ]
     9. Spine [ ]
     10. Ankle dislocation [ ]
     11. Knee dislocation [ ]
     12. Elbow dislocation[ ]
     13. Shoulder dislocation[ ]
  2. Other Trauma Associated injuries (Non-orthopaedic) (ICD version 10)

……………………………………………………………………………………………………

1. Do you have any co-morbidities? Yes [ ] No [ ]
   1. If yes, which ones? (ICD version 10) ………………………………………………..

Are you currently on medications...........................................................................
